# Biogenic nano-magnetite and nano-zero valent iron treatment of alkaline Cr(VI) leachate and chromite ore processing residue

**DOI:** 10.1016/j.apgeochem.2014.12.001

**Published:** 2015-03

**Authors:** Mathew P. Watts, Victoria S. Coker, Stephen A. Parry, Richard A.D. Pattrick, Russell A.P. Thomas, Robert Kalin, Jonathan R. Lloyd

**Affiliations:** a School of Earth, Atmospheric and Environmental Sciences and Williamson Research Centre for Molecular Environmental Science, The University of Manchester, Manchester M13 9PL, UK; b Diamond Light Source, Chilton, Didcot, Oxfordshire OX11 ODE, UK; c Parsons Brinckerhoff, Queen Victoria House, Redland Hill, Bristol BS6 6US, UK; d Department of Civil and Environmental Engineering, University of Strathclyde, Glasgow G1 1XJ, UK

## Abstract

Highly reactive nano-scale biogenic magnetite (BnM), synthesized by the Fe(III)-reducing bacterium *Geobacter sulfurreducens*, was tested for the potential to remediate alkaline Cr(VI) contaminated waters associated with chromite ore processing residue (COPR). The performance of this biomaterial, targeting aqueous Cr(VI) removal, was compared to a synthetic alternative, nano-scale zero valent iron (nZVI). Samples of highly contaminated alkaline groundwater and COPR solid waste were obtained from a contaminated site in Glasgow, UK. During batch reactivity tests, Cr(VI) removal from groundwater was inhibited by ∼25% (BnM) and ∼50% (nZVI) when compared to the treatment of less chemically complex model pH 12 Cr(VI) solutions. In both the model Cr(VI) solutions and contaminated groundwater experiments the surface of the nanoparticles became passivated, preventing complete coupling of their available electrons to Cr(VI) reduction. To investigate this process, the surfaces of the reacted samples were analyzed by TEM-EDX, XAS and XPS, confirming Cr(VI) reduction to the less soluble Cr(III) on the nanoparticle surface. In groundwater reacted samples the presence of Ca, Si and S was also noted on the surface of the nanoparticles, and is likely responsible for earlier onset of passivation. Treatment of the solid COPR material in contact with water, by addition of increasing weight % of the nanoparticles, resulted in a decrease in aqueous Cr(VI) concentrations to below detection limits, via the addition of ⩾5% w/w BnM or ⩾1% w/w nZVI. XANES analysis of the Cr *K* edge, showed that the % Cr(VI) in the COPR dropped from 26% to a minimum of 4–7% by the addition of 5% w/w BnM or 2% w/w nZVI, with higher additions unable to reduce the remaining Cr(VI). The treated materials exhibited minimal re-mobilization of soluble Cr(VI) by re-equilibration with atmospheric oxygen, with the bulk of the Cr remaining in the solid fraction. Both nanoparticles exhibited a considerable capacity for the remediation of COPR related Cr(VI) contamination, with the synthetic nZVI demonstrating greater reactivity than the BnM. However, the biosynthesized BnM was also capable of significant Cr(VI) reduction and demonstrated a greater efficiency for the coupling of its electrons towards Cr(VI) reduction than the nZVI.

## Highlights

• Remediation of high pH Cr(VI) contamination related to COPR and its groundwater.
• Extensive characterization of COPR and its related groundwater.
• Cr(VI) reduction to Cr(III) by biogenic nano-magnetite and nano-zero valent iron.
• Critical assessment of reactivity and passivation using spectroscopy and imaging.
• Stabilization of the COPR Cr(VI) source by addition of nanoparticles.

## 1 Introduction

Chromium is a priority pollutant found at contaminated sites associated with a variety of previous land uses; from its initial mining and processing as a chromite ore (Kimbrough et al., 1999) through to its use in metallurgy, leather tanning, pigment production and wood preservatives (Kamaludeen et al., 2003). Cr is redox sensitive, and under environmental conditions, typically occurs in the Cr(III) and Cr(VI) oxidation states. The oxidation state exerts a major control over its physicochemical and biochemical behavior (Kotaś and Stasicka, 2000). Cr(VI) typically occurs as the hydrolyzed species; H_2_CrO_4_, HCrO_4_^−^, CrO_4_^2−^, and Cr_2_O_7_^2−^, which are readily soluble and weakly adsorb to mineral surfaces (Kimbrough et al., 1999), causing oxidative damage upon entering the cell (Dayan and Paine, 2001). Cr(VI) therefore behaves as an irritant, carcinogen and allergen (Chen and Hao, 1998). Due to its toxicity the World Health Organization has set an upper limit of 0.05 mg L^−1^ for Cr(VI) in drinking water (WHO, 2008). By contrast, Cr(III) is an essential trace nutrient necessary for glucose and lipid metabolism (Wang, 2000). The Cr(III) oxidation state is much less mobile, typically forming relatively insoluble Cr oxy-hydroxides, considered to be recalcitrant to re-oxidation by dissolved oxygen (Rai et al., 1989) or strongly adsorbing to mineral surfaces (Fendorf, 1995). Other more oxidizing species found in waters and soils, such as Mn(IV) oxides, are capable of Cr(III) re-oxidation (Fendorf and Zasoski, 1992). This is however considered a dissolution controlled reaction and is kinetically slow under most environmental conditions (Rai et al., 1989).

Extensive Cr(VI) contamination has occurred within the urban area of Glasgow, UK, which was a centre for chromite ore processing until closure of the chemical works responsible in 1968 (Farmer et al., 1999, 2006). A high lime processing technique was used to extract Cr from chromite ore, involving heating the ore with lime, thereby oxidizing insoluble Cr(III) to the soluble Cr(VI), followed by aqueous leaching (Burke et al., 1991). Globally the “high lime” technique met 40% of the demand for Cr processing in 2001, principally in Eastern Europe, India, Pakistan and China (Darrie, 2001), the disposal of this waste is also responsible for Cr(VI) contamination issues (Cheng et al., 2012). In Glasgow this technique produced >2 million tonnes of chromite ore processing residue (COPR), a cement like material with appreciable quantities of remnant Cr(VI) (Farmer et al., 2002). This material was subsequently used as construction material or backfilled into quarries, principally in the South East of Glasgow. In these areas, soil Cr concentrations have been found to be in the range of thousands of mg kg^−1^ (Fordyce et al., 2012), with soluble Cr(VI) concentrations in ground and surface waters up to 100 mg L^−1^ (Farmer et al., 2006; Whalley et al., 1999). The COPR typically has a strongly buffered solution pH of between 11 and 12.5 and a total Cr content of 3–7%, with Cr(VI) reported between 1% and 30% of total Cr (Geelhoed et al., 2003).

Conventional COPR remediation techniques include physical removal, soil washing and containment strategies (CLAIRE, 2007). However, considering the large quantities of material, and the possibility of extraction leading to the risk of exposure by inhalation (Rowbotham et al., 2000), stabilization at the source is potentially a preferred option. Chemical stabilization focuses upon the reductive precipitation from Cr(VI) to Cr(III) via addition of aqueous reducing agents such as Fe(II) (Dermatas et al., 2006; Fendorf and Li, 1996) or reduced sulfur species (Graham et al., 2006; Kim et al., 2001; Wazne et al., 2007). These sulfur-containing chemical stabilization methods have however problematic aspects, principally the precipitation of secondary minerals with expansive swelling properties (Moon et al., 2007). Aqueous Fe(II) chemical stabilization has also led to rapid precipitation of Fe(II) at high pH, thus limiting Cr(VI) reduction (He et al., 2004). Alongside this anion exchange with the conjugate SO_4_^2−^ anion can occur, causing Cr(VI) release not removal (Geelhoed et al., 2003).

Particulate Fe material has been employed previously as a treatment for Cr(VI) in a variety of studies (Blowes et al., 2000, 1997; Fuller et al., 2013; Wilkin et al., 2005). The development of methods for production of Fe(0) nanoparticles (NPs) has enabled their consideration for a variety of remediation processes (Zhang, 2003). These particles, due to the significantly higher surface area, exhibit much greater reactivity than their micron-scale equivalents (Hochella et al., 2008), and can be targeted towards *in situ* source stabilization (Ponder et al., 2000). The reduction of Cr(VI) by Fe NPs, primarily at acidic to near neutral test conditions, has therefore been the subject of several studies using Fe(0) (Manning et al., 2006; Ponder et al., 2000) and magnetite (Cutting et al., 2010; Yuan et al., 2009). Their use in treatment of high pH groundwater and stabilization of COPR has also been proposed as an alternative to aqueous chemical stabilization (Cao and Zhang, 2006; Du et al., 2012; Li et al., 2008).

A further development in this area has followed earlier findings that microbes can produce reactive phases that can be harnessed for the transformation of inorganic contaminants (Lovley, 1995). While direct enzymatic microbial Cr(VI) reduction has been studied intensively in laboratory cultures, its transformation in more complex environmental systems is often mediated by indirect interactions with biogenic Fe(II) (Fendorf et al., 2000) formed by dissimilatory Fe(III)-reducing bacteria (Hansel et al., 2003; Wielinga et al., 2001). This process has also been identified as a likely mechanism for Cr(VI) reduction *in situ* in soils adjacent to COPR deposits (Stewart et al., 2010, 2007; Whittleston et al., 2011a, 2011b, 2012). However, the pH values of 10–11 represent the known upper limits for alkaliphilic Fe(III) reduction (Pollock et al., 2007; Rizoulis et al., 2012; Williamson et al., 2013; Ye et al., 2004), while the pH of COPR is typically >12 (Geelhoed et al., 2002). There is a clear need for a viable *in situ* treatment of COPR that does not require prior acidification, which would be problematic due to the high acid neutralization capacity of the material (Tinjum et al., 2008). One possible approach that warrants testing is the optimized synthesis of biogenic Fe(II) reactive phases, for example nano-scale magnetite, in a carefully controlled bioreactor system (Byrne et al., 2011; Cutting et al., 2009), which could enable efficient production and subsequent application of the biogenic nanoparticles (NPs) for remediation (Cutting et al., 2010; Telling et al., 2009).

The over-arching aims of this study were to assess and compare the applicability of biogenic nano-magnetite (BnM), synthesized by the microbial reduction of Fe(III), and commercially available synthetic nano-scale zero valent iron (nZVI) for the remediation of high pH aqueous Cr(VI) and the stabilization of Cr(VI) within COPR. The rate of Cr(VI) removal and the stoichiometry of the reaction in aqueous pH 12 model solutions are compared to those in contaminated groundwater; to assess any impact of the more chemically complex environmental system. This will specifically address the role of passivation in limiting the reactivity of the NPs, and employ surface sensitive analytical techniques to probe the surface chemistry of the reacted NPs. The ability to effectively stabilize the soluble Cr(VI) fraction of the COPR will be assessed, exploring the accessibility of the Cr(VI) for reduction and its susceptibility to re-oxidation after stabilization.

## 2 Experimental section

### 2.1 Magnetite production

BnM was synthesized by the dissimilatory reduction of 2-line ferrihydrite by a culture of *Geobacter sulfurreducens* according to the method detailed in (Cutting et al., 2009). First an amorphous ferrihydrite suspension was produced by precipitation from a Fe(III)Cl_3_·6H_2_O solution by raising the pH to 7 via addition of 10 M NaOH (Lovley and Phillips, 1986). The ferrihydrite suspension was subsequently washed 6 times using 18.2 MΩ water to remove Cl^−^ ions. The total Fe concentration was then determined via extraction for 1 h of a known volume of ferrihydrite slurry, using 3 M HCl, and analyzed by the ferrozine assay (Stookey, 1970).

A culture of *G. sulfurreducens* was grown in modified freshwater medium, with 20 mM acetate and 40 mM fumerate, as electron donor and acceptor respectively, at 30°C under anaerobic conditions (Lloyd et al., 2003). Working under an atmosphere of N_2_–CO_2_ (80:20) the culture was harvested in its late log phase by centrifugation (Sigma 6k15 at 5000*g* for 20 min) and washed three times using 30 mM NaHCO_3_ at pH 7.

The washed cells were then added to make an optical density in the final incubation, at 600 nm, of 0.4, to autoclaved serum bottles containing an anoxic solution of 20 mM Na acetate, 50 mM ferrihydrite, 30 mM NaHCO_3_ and 10 μM AQDS. The serum bottles were then incubated at 30°C in the dark for 1 week. The resulting BnM was washed 3 times, to remove bacterial cells, by separating the magnetic biomineral particles with a magnet, removing the supernatant and replacing with ultrapure water (18.2 MΩ). The BnM was stored as a suspension under an anoxic atmosphere in sealed serum bottles until use.

### 2.2 Nano zero valent iron

A sample of NANOFER 25S nZVI was obtained from NANO IRON, s.r.o., Czech Republic, consisting of a stabilized aqueous dispersion of Fe(0) NPs. The nZVI was received in sealed containers and stored unopened at 4°C until use.

### 2.3 Chromite ore processing residue and Cr(VI) contaminated groundwater

A sample of contaminated groundwater was taken from a monitoring well within a COPR waste site as part of the M74 motorway expansion project in the south east of Glasgow, UK. The unfiltered water was transferred to a clean sterile container and stored in the dark at a temperature of 10°C until use.

COPR was obtained from a different site also in the south east of Glasgow, which received large quantities of material from the Rutherglen Chromite Processing Works. The sample was taken from a waste fill site where the material was extracted from an exploratory borehole during the M74 motorway expansion project. The pale granular material was tentatively identified as COPR from the presence of a high pH yellow leachate, characteristic of chromate solutions. Roughly 2 kg of the field moist COPR material was transferred to a sterile polythene bag and stored in the dark, loosely double bagged at 10°C until use.

### 2.4 Wet chemical analysis

Fe(II) and Fe(total) concentrations were determined by acid extraction and the spectrophotometric ferrozine assay (Stookey, 1970). Samples were extracted using 0.5 M HCl for 1 h to determine the readily extractable Fe(II). For Fe(total) measurements an additional reduction step was undertaken by reaction with the reductant hydroxylamine hydrochloride (6.25 M) for 24 h. The Fe(II) concentration was then determined by reaction with the ferrozine solution (Lovley and Phillips, 1986). Aqueous Cr(VI) concentrations were determined by a spectrophotometric assay with 1,5-diphenylcarbazide (DPC) (Skougstad et al., 1979). All UV–VIS measurements were performed using a Jenway 6715 UV/Vis spectrophotometer and compared to calibration standards on the day of measurement.

The pH and redox potentials of the COPR slurry samples were recorded using a bench top meter (Denver Instrument UB-10) and a calibrated probe (pH: P Cole Parmer 5990-45 CCP, Eh: Mottler Toledo InLab Redox Micro).

An alkaline digestion and extraction technique was used to determine the total bulk Cr(VI) content of the COPR sample (EPA, 1992). A 1 g dry weight equivalent sample of the COPR was digested using 50 mL of a 0.28 M Na_2_CO_3_/0.5 M NaOH solution heated to 90–95°C for 60 min. The cooled mixture was filtered through a 0.45 μm filter and pH adjusted to 7.5 using 5 M nitric acid. A sample of the filtrate was then analyzed using the DPC method.

### 2.5 Analytical techniques

The specific surface area of the BnM and nZVI was obtained using the Brunauer, Emmett and Teller (BET) N_2_ adsorption method (Brunauer et al., 1938). Analysis was conducted using a Micromeritics Gemini instrument and the adsorption/desorption isotherms were recorded using helium at the temperature of liquid nitrogen (77 K). All samples were dried prior to analysis and degassed using He at 40°C for 24 h (*MicromeriticsFlowprep 060*). Finally, specific surface area was calculated by the multipoint analysis method.

Inductively Coupled Plasma Atomic Emission Spectroscopy (ICP-AES) was performed on a Perkin–Elmer Optima 5300 dual view ICP-AES. Prior to analysis all samples were 0.22 μm filtered and diluted to 2% HNO_3_.

Ion Chromatography (IC) was performed on a Metrohm 761 Compact IC with a Metrohm 813 autosampler using a conductivity detector.

Transmission electron microscopy (TEM) analysis was carried out using a FEI Tecnai TF20 microscope, at an operating beam voltage of 200 kV, equipped with an energy dispersive X-ray analysis (EDX) system (Oxford Instruments INCA 350/80 mm X-Max SDD detector), field emission gun (FEG), high angle annular dark field (HAADF) detector, and a GatanOrius SC600A CCD camera. Samples were prepared by dispersion in ethanol and a droplet placed upon a carbon grid (Agar Scientific, Stansted, UK) before drying prior to analysis.

X-ray diffraction (XRD) measurements were carried out using a Bruker D8 Advance diffractometer fitted with a Göbel mirror and using a Cu Kα1 X-ray source. Diffraction data were obtained in 2*θ* geometry over a range of 5–70° consisting of 0.02° steps and a 2 s counting time.

X-ray fluorescence (XRF) data were analyzed using an AxiosPANalytical spectrometer with Omnian standardless analysis for major element analysis and classic Pro-Trace software for trace element analysis. Prior to analysis the sample was dried at 110°C and a loss on ignition at 1100°C was not performed; the results therefore reflect the composition minus water and carbonate.

X-ray photoelectron spectroscopy (XPS) analysis of the un-reacted and the model Cr(VI) solution reacted BnM and nZVI were conducted using a VG Escalab 250 instrument employing a monochromatic Al Kα X-ray source and an analyzer pass energy of 20 eV, with a total energy resolution of ∼0.9 eV. A flood gun was used to expose the photoemitting surface to low energy electrons in order to create uniform charge neutralization. The XPS spectra of aerobic and anaerobic contaminated groundwater reacted BnM and nZVI were obtained using a Kratos Axis Ultra spectrophotometer. Employing a monochromated Al Kα X-ray source and an analyzer pass energy of 80 eV (wide scans) and 20 eV (narrow scans) with a total energy resolution of 1.2 and 0.6 eV respectively. Both systems were carried out at a system base pressure of 5 × 10^−10^ mbar. The photoelectron binding energies (BE) reported for all samples are referenced to the C 1s adventitious carbon peak set at 285 eV BE. A Shirley background was employed during fitting of the data (Shirley, 1972). The Fe 2p region was subject to fitting of GS multiplets (Gupta and Sen, 1975), surface structures and shake-up features (Grosvenor et al., 2004), using components comprising of 70% Lorentzian and 30% Gausian (McIntyre and Zetaruk, 1977).

X-ray absorption spectroscopy (XAS) analysis of the Cr *L*_2,3_ and Fe *L*_2,3_ edge were conducted on beamline 4.0.2 at the Advanced Light Source (ALS), Berkeley, CA, and X-ray magnetic circular dichroism (XMCD) spectra derived using the octopole magnet endstation (Arenholz and Prestemon, 2005). The ALS runs in top up mode with a ring current of 500 mA and an energy of 1.9 GeV. Energy calibration at the Fe *L*_3_ edge was achieved using a standard natural magnetite and Fe foil. Total electron yield (TEY) mode was used to monitor the X-ray absorption (XA) of the sample, giving a probing depth of ∼4.5 nm. By reversing the 0.6 T applied field photon energies the XA were measured with circularly polarized X-rays for the two opposite magnetization directions. The spectra were subsequently normalized to the incident beam intensity and subtracted to give the XMCD spectrum (Pattrick et al., 2002).

Cr *K* edge XAS spectra were recorded in transmission mode on Beamline B18 at the Diamond Light Source (DLS) operating in a 10 min top-up mode for a ring current of 250 mA and an energy of 3 GeV. The radiation was monochromated with a Si(1 1 1) double crystal and harmonic rejection was achieved through the use of two platinum-coated mirrors operating at an incidence angle of 7.0 mrad. The monochromator was calibrated using the *K* edge of Cr foil; taking the first inflection point as 5989 eV. Samples were maintained under anoxic conditions and cooled using a LN_2_ cryostat (Oxford Instruments, Optistat DN2) with PT100 sensor integration into the sample holder. X-ray absorption near edge structure (XANES) data were analyzed using ATHENA software (Ravel, 2009). The individual scans were merged and background subtracted. The data were then subject to linear combination fitting (Kelly et al., 2008), in ATHENA, to determine the Cr(VI) to Cr(III) ratio, using K_2_CrO_4_, Cr(VI), and FeCr_2_O_4_, Cr(III), standards measured during the same beamtime as the samples.

### 2.6 Batch NP treatment of aqueous Cr(VI)

Batch experiments were performed in triplicate in acid washed 120 mL serum bottles containing 100 mL of Cr(VI) contaminated groundwater, or a known concentration of model Cr(VI) solution (K_2_CrO_4_), using ultrapure water adjusted to pH 12 with 10 M NaOH. The serum bottles were sealed using butyl rubber stoppers and aluminum crimps, and autoclaved to 121°C to ensure sterility. Anoxic replicates were degassed using pressurized N_2_ gas passed through a 0.22 μm filter, while aerobic replicates were equilibrated with atmospheric air. Prior to addition of the NP, a sample was removed to determine the background Cr(VI) value. Samples were removed from the serum bottles using an N_2_ degassed syringe, 1 mL of the solution being removed for Cr(VI) analysis. A known addition of either BnM or nZVI was then added to the serum bottles. Between sampling the serum bottles were maintained in the dark at 20°C on a rotating shaker. The pH of the solution was recorded at the end of the experiment. After incubation the NPs were separated magnetically and washed using N_2_ degassed ultrapure water. The washed samples were then dried under an N_2_ atmosphere for analysis.

### 2.7 Evaluation of aqueous Cr(VI) removal kinetics

The aqueous Cr(VI) removal data from batch experiments was described using a pseudo-1st order kinetic model:

$$\frac{\text{d}[\text{Cr}(\text{VI})]}{\text{d}t}=-k_{\text{obs}}[\text{Cr}(\text{VI})]$$

where [Cr(VI)] represents the concentration of aqueous Cr(VI), *t* time and *k*_obs_ the respective rate constant.

### 2.8 Stabilization of Cr(VI) associated with COPR by NP addition

Batch microcosm experiments containing 25 g of field moist COPR and 50 mL ultrapure water were prepared in 120 mL serum bottles in duplicate. These were then degassed using pressurized N_2_ gas passed through a 0.22 μm filter. Increasing additions (as % by mass of field wet COPR) of the relevant NP, BnM or nZVI, as an aqueous dispersion, were made to the microcosms. The bottles were homogenized by shaking and stored in the dark at 20°C. At regular intervals, slurry samples were removed, using an N_2_ degassed syringe, and analyzed for various geochemical parameters. At 50 days of incubation, slurry samples were removed and dried under an N_2_ atmosphere for XANES and XRD analysis.

### 2.9 Re-oxidation of NP treated COPR

After 100 days, 25 mL of the COPR material was removed from the above experiment and transferred into 250 mL Erlenmeyer flasks. The flasks were exposed to atmospheric air, stoppered with cotton wool and sealed with Parafilm to stop evaporation and stored in the dark at 20°C in a shaking incubator set to 100 rpm. To ensure the maintenance of atmospheric oxygen within the flasks, the Parafilm was removed every 2–4 days to allow equilibration of the headspace with atmospheric air. Again slurry samples were removed at regular intervals and analyzed for various geochemical parameters.

## 3 Results and discussion

### 3.1 Initial NP characterization

Prior to testing the biogenic and synthetic NPs against Cr(VI), they were analyzed using a range of techniques. XRD data identified magnetite as the only detectable crystalline phase present in the microbially synthesized BnM sample, while the nZVI had a mixed Fe(0) and magnetite composition, see Supporting information Fig. SI 1. Magnetite is a common corrosion product of Fe(0) (Furukawa et al., 2002), especially under anoxic conditions, and is often reported in a shell like structure on the surface of un-reacted nZVI particles (Li et al., 2006). BET specific surface areas of 17.1 m^2^ g^−1^ and 14.6 m^2^ g^−1^ were recorded for the BnM and nZVI respectively. These figures should be regarded as a conservative estimate due to significant aggregation, and are therefore not used to interpret reaction rates.

### 3.2 Groundwater characterization

Approximately 94% of the aqueous Cr was present as the Cr(VI) valence state in groundwater from the COPR-contaminated field site, consistent with the poor solubility of Cr(III) at the alkaline pH of 11.9 (Kimbrough et al., 1999). The major chemical component was SO_4_^2−^, Table 1, which was observed at higher concentrations than previously reported values for COPR related groundwater (Farmer et al., 2006). The other significant component, Ca, is likely a reflection of the calcareous nature of the COPR from which the groundwater was sourced (Farmer et al., 2002). It should be noted that the chemical data presented have a significant charge imbalance, and although not analyzed for, the presence of other cations such as Na^+^ would potentially account for this.

### 3.3 Aqueous Cr(VI) removal – reaction stoichiometry and rates

**Fig. 1:**
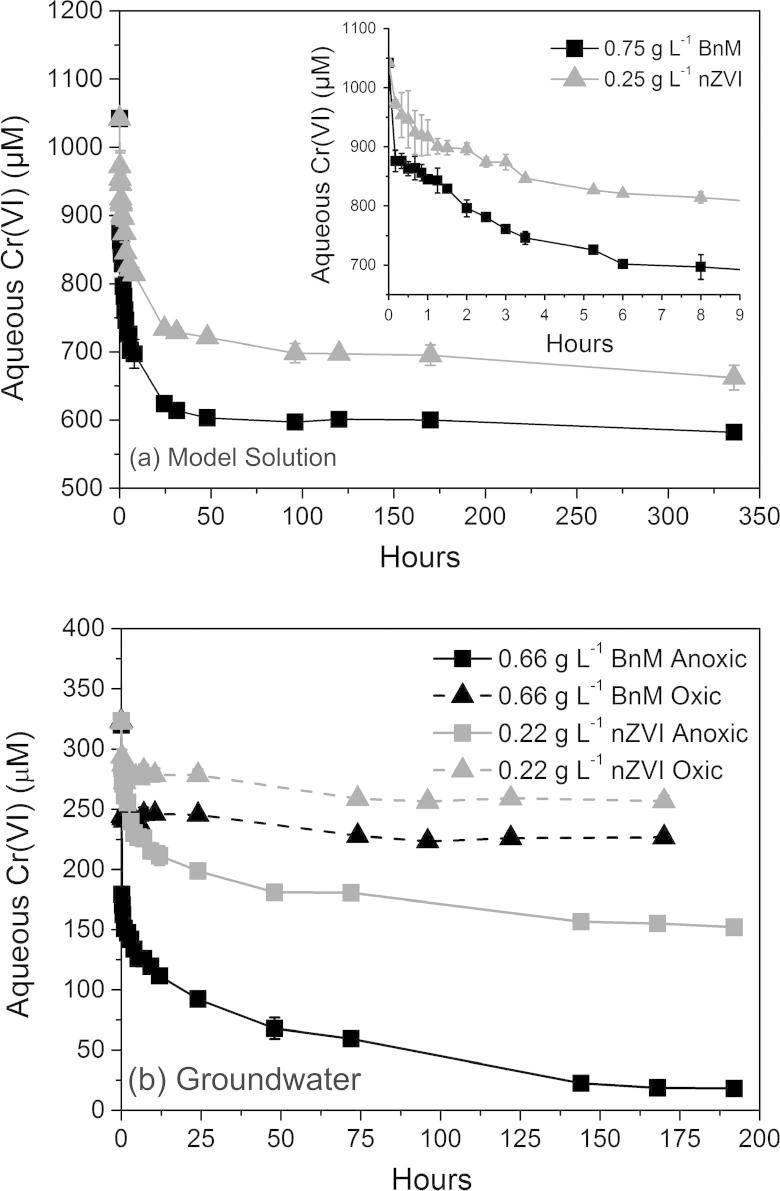
Aqueous Cr(VI) removal from a model pH 12 Cr(VI) solution (a) and a Cr(VI) contaminated groundwater (b) over time, when amended with BnM or nZVI. Inset graph (a) shows removal over the first 9 h of the reaction. Error bars represent the standard deviation of the triplicate values. Note the different scale bars used in the individual plots.

Both NPs showed rapid removal over the initial 24 h of reaction upon amendment of model Cr(VI) solutions or groundwater with nZVI or BnM, followed by a loss in reactivity despite the presence of further aqueous Cr(VI), see Fig. 1a and b. The presence of oxygen in the groundwater was observed to significantly inhibit Cr(VI) removal by both NPs (Fig. 1b). No Cr(III) was found to be present in solution (by subtracting soluble Cr(VI) values determined by the DPC assay from total soluble Cr values by ICP-AES; data not shown), and is therefore most likely to have been precipitated as insoluble Cr(III) (Rai et al., 1989).

The initial Cr(VI) removal rate from the Cr(VI) solutions (Fig. 1) was modeled as a pseudo-1st order reaction and the respective rate constants (*k*_obs_) calculated from data presented in Supporting information Fig. SI 2. The data were fitted between the first data point after addition of the NPs and up to 315 min, where a linear regression correlation coefficient (*R^2^*) of ⩾0.90 was recorded, data presented in Table 2. In all experiments the pH remained within the starting value of 12 ± 0.2 and therefore is not thought to have impacted upon removal rates. Assuming a linear relationship between mass additions and the rate of Cr(VI) removal, the *k*_obs_ values can be normalized per gram of NP (*k*_mass_), again presented in Table 2. The *k*_mass_ values of the nZVI are, in both model solutions and in contaminated groundwater, approximately double those recorded using BnM. This is possibly a reflection of the greater reactivity posed by nZVI compared to BnM. Interestingly the *k*_mass_ values calculated from Cr(VI) removal data upon reaction with groundwater are higher than those from the geochemically simpler model solutions. The reason for this increase in reaction rate is unclear, but is potentially due to different reactive surface area (dependent upon mass addition) to Cr(VI) ratio used in the two experiments. Although the reducing species of the NPs used are initially in excess, the ability to remove Cr(VI) is evidently exhausted, potentially causing a deviation from true pseudo-1st order behavior.

After initial rapid Cr(VI) removal a plateau of minimal further removal occurred, this was taken as the maximum Cr(VI) removal per mass addition of NP, presented in Table 2. The nZVI caused greater removal of Cr(VI) from solutions, per g of NP, removing approximately double the mass removed by BnM in all of the experimental conditions. Maximum removal occurred in the anoxic pH 12 model solutions for both NPs, with a decrease in removal reported when reacted in the more chemically complex groundwater. The removal of Cr(VI) from COPR groundwater by nZVI reported here is below that previously reported in (Cao and Zhang, 2006), of 84–109 mg g^−1^ Fe(0). This difference in removal capacity could be accounted for by the employment of different NP synthesis techniques resulting in different reactive properties, or differences in the chemical composition of the groundwater. The most significant inhibition for both NPs was noted for Cr(VI) removal from groundwater in the presence of atmospheric oxygen, with 72% and 63% inhibition compared to anoxic conditions, for BnM and nZVI respectively.

To further probe the efficiency of the coupling of reducing species to Cr(VI) it is worth considering the stoichiometry of the respective reactions. When considering stoichiometric reduction of 1 mol of Cr(VI) to Cr(III), 3 mol of Fe(II) or 1 mol of Fe(0) would be required, where the principle reductant in the BnM is Fe(II) and in the nZVI, Fe(0), via the half reactions:

$$\text{Cr}(\text{VI})+3{\text{e}}^{-}\to \text{Cr}(\text{III})$$

$$3\text{Fe}(\text{II})-3{\text{e}}^{-}\to 3\text{Fe}(\text{III})$$

or

$$\text{Fe}(\text{0})-3{\text{e}}^{-}\to \text{Fe}(\text{III})$$

The nZVI therefore has a greater number of electrons available for donation per mole, by the Fe(0), compared to the Fe(II) of BnM. This is combined with the greater density of the reducing agent, Fe(0), in the nZVI particles, where it represents ∼85% of total Fe by mass, as characterized by the supplier. In comparison, the BnM, with one third of the iron in the ferrous oxidation state in the Fe_3_O_4_ structure, contains only 24% Fe(II) by mass.

Considering the above factors, the maximum theoretical electron donating capacity (EDC), as mol electrons per g of NP, for the nZVI and BnM, have been calculated and are presented in Table 2. These values demonstrate the greater reducing potential of the nZVI, which has nearly 10 times the number of electrons for donation compared to the BnM. The number of electrons consumed by the reduction of Cr(VI) to Cr(III), and the % this represents of the maximum EDC, for each experiment, are also presented in Table 2. The % of electrons consumed, compared to available (EDC), prior to loss of reactivity, was below those expected for complete oxidation of the reductant under all reaction conditions. Reduction of an aqueous species by a solid phase reductant is widely accepted to be a surface process, where the aqueous species adsorbs allowing a transfer of electrons (Manning et al., 2006). A key process in the incomplete transfer of electrons from the NP to the Cr(VI) is by passivation of the reactive surface (Peterson et al., 1997). This occurs where the reactive sites at the particle surface, either Fe(II) or Fe(0), become oxidized to Fe(III) or are coated with precipitates forming an insulating layer, preventing contact and further electron transfer to aqueous Cr(VI). This has been reported previously for magnetite reacted with Cr(VI) at neutral (Peterson et al., 1996, 1997) and high pH (He and Traina, 2005) conditions. The lower % of electrons consumed by Cr(VI) in anoxic groundwater is potentially due to the more complex chemistry of the solution, where the adsorption of groundwater components upon the reactive surface could prevent further contact with Cr(VI). The presence of additional groundwater components has been implicated previously in the inhibition of Cr(VI) removal by Fe(0) (Lo et al., 2006), see Section 3.4 for further discussion. The inhibition of Cr(VI) reduction in the presence of atmospheric oxygen is inferred to be due to its behavior as a known oxidant of Fe(II) and Fe(0) (Fendorf, 1995), competing directly with Cr(VI) for reactive surface sites (Eary and Rai, 1988).

Despite lower overall Cr(VI) removal, the BnM system is able to couple more of its available electrons (EDC) to the reduction of Cr(VI) than the nZVI. This is likely a manifestation of the smaller size, as discussed with TEM results in Section 3.4, and its inferred greater surface area. A higher specific surface area would increase the number of available reactive Fe sites upon the surface, per mass of NP, for electron exchange reactions with Cr(VI) (Ponder et al., 2001). In addition, when considering the nZVI system, the concurrent process of anoxic corrosion by H_2_O, producing H_2_ gas, also consumes electrons (Furukawa et al., 2002), in direct competition with Cr(VI). The identification of magnetite, by XRD, would support some oxidation from Fe(0) to Fe(II) and Fe(III). This can account for some of the inhibition of Cr(VI) reduction observed in the nZVI and the lower % of its EDC consumed by Cr(VI) reduction.

A further potential limitation on the efficient electron transfer between Cr(VI) and the NP is the prevalent alkaline pH, which has also previously been implicated in inhibition of Fe(II) mediated Cr(VI) reduction (He et al., 2004). This is due to electrostatic repulsion of the negative CrO_4_^2−^ with the also negative Fe–O^−^ surface phase, which dominate high pH environments above the point of zero charge; which for magnetite and Fe(0) is typically between 6 and 8 (Fendorf, 1995; Kosmulski, 2004; Rai et al., 1989; Sun et al., 2006). However, the assessment of the impact of this upon Cr(VI) removal, compared to neutral solutions, is beyond the scope of this study.

### 3.4 Characterization of un-reacted and aqueous Cr(VI) reacted NPs

**Fig. 2:**
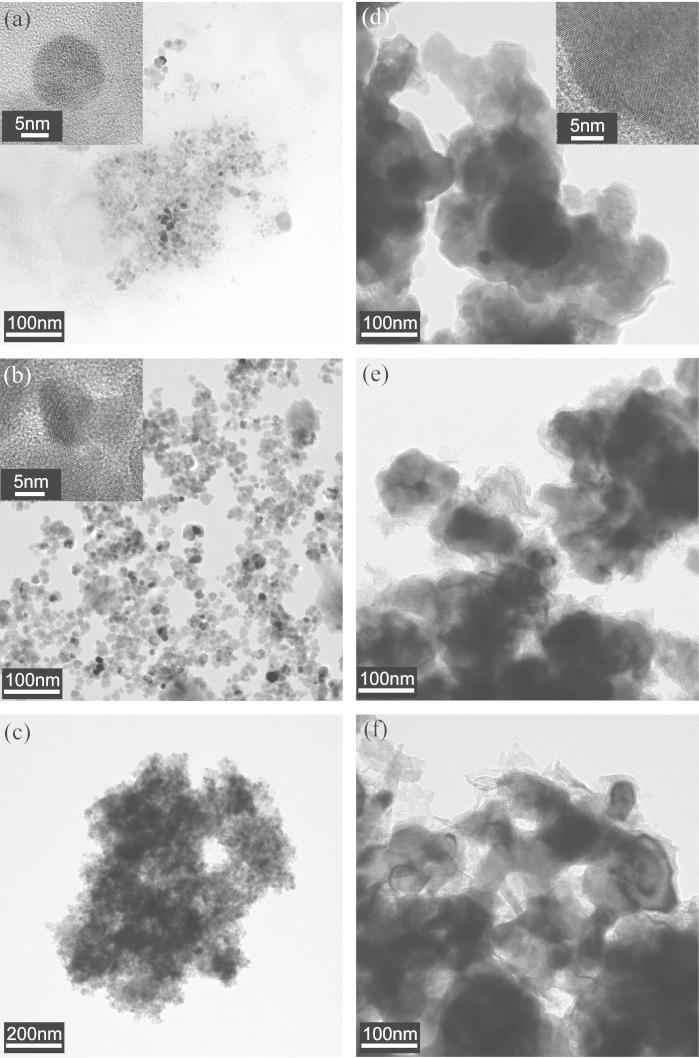
TEM images of un-reacted BnM (a) and nZVI (d), model Cr(VI) solution reacted BnM (b) and nZVI (e) and groundwater reacted BnM (c) and nZVI (f).

The NPs, both before and after reaction with Cr(VI) in model test solutions or contaminated groundwater samples, were analyzed using a range of imaging and spectroscopy techniques. Upon TEM analysis (Fig. 2) of the un-reacted BnM particles, it is evident they were visibly smaller in diameter (5–20 nm) than the larger nZVI particles (40–100 nm). Both, however, displayed degrees of particle aggregation. No obvious morphological change, upon Cr(VI) reaction, could be observed in either NP.

**Fig. 3:**
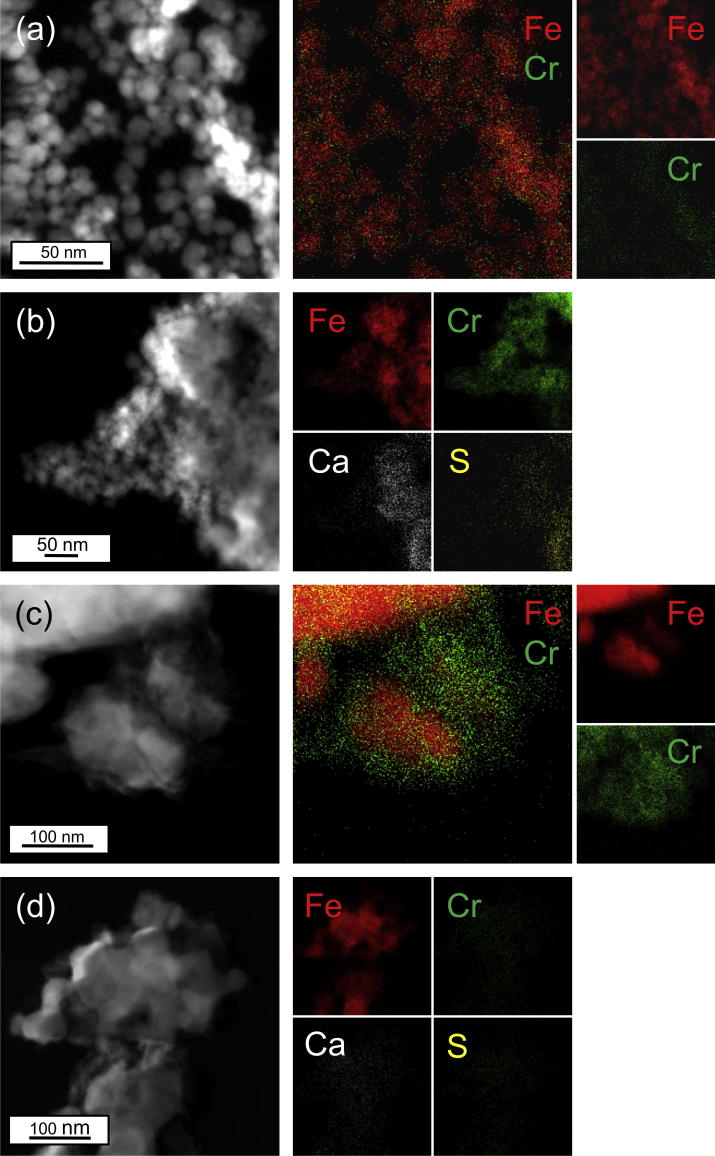
TEM HAADF images and their corresponding TEM-EDX elemental maps for; (a) BnM reacted with a model Cr(VI) solution, (b) BnM reacted with Cr(VI) contaminated groundwater, (c) nZVI reacted with a model Cr(VI) solution and (d) nZVI reacted with Cr(VI) contaminated groundwater.

The HAADF and corresponding TEM-EDX of the model solution reacted BnM particle surfaces (Fig. 3a), observed a strong positive association of Cr with Fe abundance, consistent with Cr being retained by the BnM surface (Telling et al., 2009). In contrast the Cr(VI) reacted nZVI appeared to have an overgrowth of a Cr rich and Fe poor structure, alongside an overlapping Fe and Cr abundance (Fig. 3c). This is assumed to be related to the greater Cr(VI) removal of the nZVI and saturation of its surface with Cr causing a thicker Cr accumulation on the particle surface. The groundwater reacted NPs (Fig. 3b and d) showed similar Cr relationships to those observed with the model solutions, with the addition of the presence of groundwater components Ca and S.

**Fig. 4:**
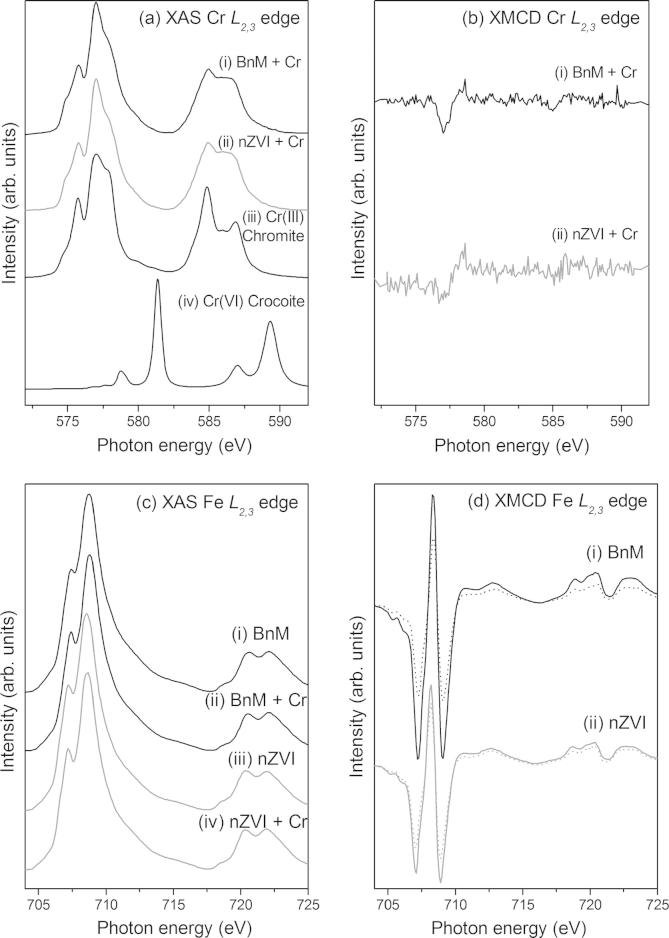
(a) Cr *L*_2,3_ edge XAS spectra of Cr(VI) reacted BnM (i) and nZVI (ii) compared to standards for Cr(III), chromite, (iii) and Cr(VI), crocoite, (iv) from Kendelewicz et al. (1999). (b) Cr *L*_2,3_ edge XMCD spectra of Cr(VI) reacted BnM (i) and nZVI (ii). (c) Fe *L*_2,3_ edge XAS spectra of un-reacted BnM (i) and nZVI (iv) and Cr(VI) reacted BnM (ii) and nZVI (iv). (d) Fe *L*_2,3_ edge XMCD spectra for un-reacted (solid line) and Cr(VI) reacted (dashed line) BnM (i) and nZVI (ii).

To probe further the fate of the Cr after treatment with the NPs, the synchrotron-based spectroscopic techniques, XAS and XMCD, were used to analyze samples of BnM and nZVI before and after reaction with model Cr(VI)-contaminated solutions. Briefly, XMCD spectra are calculated from the difference between XAS spectra obtained using opposing circularly polarized light. These techniques probe the valence state of the target element and are also able to give information on the relative site occupancy of the Fe and Cr (Telling et al., 2009). The Cr *L*_2,3_ edge XAS spectra in Fig. 4, for the Cr(VI) reacted with BnM (i) and nZVI (ii), are similar in shape to the reference spectra for chromite, Fe(II)Cr(III)_2_O_4_. There is no evidence in the two spectra of a distinctive Cr(VI) peak at ∼582 eV present, as seen in the reference crocoite (PbCr(VI)O_4_) spectrum. Therefore, qualitatively, the evidence shows the Cr associated with the NPs is Cr(III), thus confirming reduction of Cr(VI) by the NPs.

The Fe *L*_2,3_ edge XAS spectra of the BnM samples (Fig. 4c) are typical of a mixed Fe(III)-rich, Fe(III)(II) spinel oxide with more Fe(III) than that expected in stoichiometric magnetite (Jiménez-Villacorta et al., 2011). The relatively high Fe(III) contribution to the XAS spectra is likely to represent an oxidized outer layer of the NPs which, as the X-ray penetration depth is only 3–4 nm, contributes a significant component of the Fe *L*_2,3_ XAS spectrum. The XAS will show a contribution from non-magnetic Fe(III) oxide layers as well as any magnetic fraction of the material (Coker et al., 2009), whereas the XMCD will only give a spectrum with contributions from magnetic material. The XMCD of the BnM (Fig. 4d) shows the characteristic three peak spectrum of magnetite where each peak reflects the three types of Fe occupancy in the structure (Pattrick et al., 2002). The three positive and negative peaks, in increasing energy, represent Fe(II) octahedral (O_h_), Fe(III) tetrahedral (T_d_) and Fe(III)O_h_ site occupancy. The Fe *L*_2,3_ edge XAS spectra of the nZVI and Cr(VI) reacted nZVI (Fig. 4c) also resemble the spectra of a mixed valence Fe phase, in contrast to that expected from a pure Fe(0) phase. The XAS spectra presented here have a main Fe *L*_3 peak_ at ∼708.5 eV with a shoulder at lower energy, compared to the featureless spectrum expected for Fe(0) with an *L*_3_ peak at ∼707 eV (Jiménez-Villacorta et al., 2011). The Fe *L*_2,3_ edge XMCD spectra from the nZVI (Fig. 4d) are also similar to the magnetite-like spectrum as seen in the BnM samples. As with the BnM samples this is interpreted as a significant amount of magnetite in the top 4.5 nm, possibly alongside an additional Fe(III) surface oxide coating (Coker et al., 2007), with no detectable contribution from the Fe(0) core. XRD characterization had revealed a magnetite component in the nZVI NPs, and from X-ray spectroscopy this appears to be concentrated in their outer layers.

The Fe *L*_2,3_ edge XAS and XMCD, Fig. 4c and d, can be used to assess the changes in relative Fe cation site occupancy, quantifying Fe(II)(O_h_), Fe(III)(T_d_) and Fe(III)O_h_ (Pattrick et al., 2002), after reaction with Cr(VI) at pH 12. In stoichiometric magnetite the site occupancy ratios are 1:1:1, and Cr substitution into magnetite will reduce the Fe occupancy in the relevant sites. In chromite the Cr(III) occupies the octahedral sites and therefore as Cr is substituted into magnetite, the Fe(III) and Fe(II) O_h_ sites are replaced. This would be manifest as a reduction in the two negative (O_h_) peaks in the XMCD spectrum (Fig. 4d). Indeed, it has been reported previously that Cr(III) occupies the Fe(II) O_h_ site in magnetite nanoparticles, causing an imbalance in the stoichiometric ratio of Fe(II) and Fe(III) in the O_h_ site (Telling et al., 2009). However, quantitative analysis of the XMCD spectra, shows that both the BnM and nZVI Fe *L*_2,3_ edge XMCD spectra show an equal concomitant loss of Fe(II) O_h_ and a gain of Fe(III) O_h_, (Table 3), suggesting oxidation of the Fe(II) in this site but not replacement by Cr(III). This suggests that there is no significant Cr(III) occupation of the Fe(II) O_h_ site upon Cr(VI) reaction at pH 12 and that the Cr(III) most likely forms a discrete phase upon the NPs surface. Support for this comes from the very weak Cr *L*_2,3_ edge XMCD signal from these samples (Fig. 4b), from this surface sensitive technique. Previous authors (Crean et al., 2012; Telling et al., 2009) have suggested weak Cr XMCD signals with magnetite nanoparticles reacted in excess Cr(VI) could be due to the saturation of Cr(III) substituted into the spinel lattice. However, the Fe *L*_2,3_ XMCD data show no evidence of this in these samples, and it is therefore interpreted to indicate the formation of a discreet non-magnetic Cr phase on the particle surface.

XPS was also used to probe the chemistry of the uppermost ∼5–10 nm of the particles (Crean et al., 2012), accounting for the majority of the BnM structure and the surface layer of the larger nZVI particles. Samples from experiments conducted with both model Cr(VI) solutions and contaminated groundwater were analyzed. The relative surface atomic % of Fe and reacted species were calculated using relative peak heights of the main component peaks in initial wide scans, subtracting O and C (Table 3). Higher % Cr was recorded for both NPs when reacted with the pH 12 model Cr(VI) solution compared to groundwater reacted samples, in accordance with higher levels of aqueous Cr(VI) removal. The groundwater reacted NPs also had a significant contribution from Si, Ca and S, totaling over 50% of the atomic % accounted for by XPS analysis, of which Ca and S were confirmed to be present in high concentrations within the groundwater. Inhibition of Cr(VI) removal by dissolved species when reacted with Fe surfaces has been noted previously in regards to Ca (Lai and Lo, 2008), sulfate (Zachara et al., 1987) and Si (Fuller et al., 2013). Their abundance on particulate ZVI barriers reacted with groundwater has also been reported (Puls et al., 1999; Wilkin et al., 2003). The association of Si, Ca and S on the surface is likely to increase passivation, and prevent Cr(VI) adsorption and reduction, accounting for the decreased Cr(VI) removal noted in the groundwater reacted NPs (compared to the model solution reacted NPs). The S component is likely to be present in the same phase as the groundwater anion, sulfate, and possibly in association with the other major cations present (Fe, Cr or Ca). Previous studies on the speciation of Ca in iron reactive barriers indicated the primary Ca phase in groundwater systems to be CaCO_3_ (Phillips et al., 2010; Wilkin et al., 2003).

**Fig. 5:**
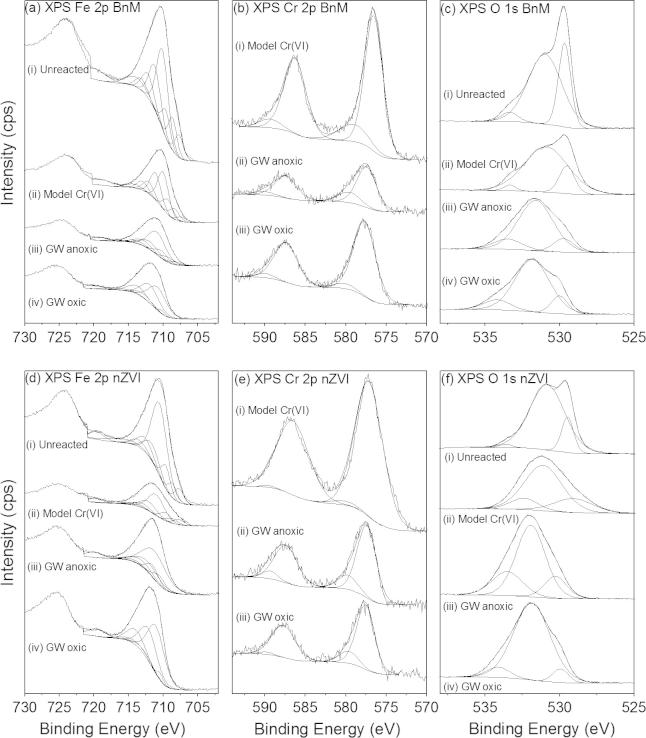
XPS spectra for the Fe 2p (a) and (d), Cr 2p (b) and (e) and O 1s (c) and (f) regions for the BnM and nZVI samples. Annotations indicate original un-reacted particles, model Cr(VI) solution reacted particles and groundwater (GW) reacted particles under anoxic or oxic conditions.

The BE of the Cr 2p_3/2_ peak (Fig. 5b and e) of the samples falls within 576.6–577.6 eV and can be fitted with primarily a single component, which is likely to be a Cr(III) phase; reported BE values for Cr(VI) are ∼579 eV and Cr(III) ∼577 eV (Aronniemi et al., 2005). In comparison to BE values reported in (Asami and Hashimoto, 1977; Biesinger et al., 2004; McCafferty et al., 1988), the lower BE value for the model solution reacted BnM, of 576.6 eV, is more comparable to Cr(III) oxides, while the slightly higher values of the other samples of 577.2–577.6 eV are more consistent with Cr(III) hydroxides. All samples, however, have an improved fit with varying minor contributions (<20%) from a Cr(VI) phase (see Table 2), suggesting an initial adsorption of the Cr(VI) prior to its reduction to Cr(III).

The Fe 2p multiplet peak fitting results for the Fe valence state (Fig. 5 and Table 3) are consistent with those obtained from XAS analysis. The un-reacted BnM Fe(II) content was slightly below values expected for stoichiometric magnetite, while the un-reacted nZVI had a lower Fe(II) % still and was lacking an Fe(0) component, that would have typically been located at ∼706.9 eV (Li et al., 2006). This suggests the presence of a partially oxidized shell over an Fe(0) core located deeper than the XPS sampling depth. Upon reaction with the Cr(VI) model solution, there was a small decrease in Fe(II) in the BnM samples, consistent with oxidation of Fe(II) coupled to Cr(VI) reduction (Jung et al., 2007; Kendelewicz et al., 2000). Reaction with anoxic groundwater caused a greater degree of oxidation of the Fe(II) of nZVI, however the groundwater component that was responsible for the increased levels of Fe(II) oxidation remain unclear. Reaction with oxic groundwater exhibited the greatest loss of Fe(II), suggesting dissolved O_2_ can access a greater proportion of Fe(II) within the NP. The BE values of the un-reacted particles of ∼710 eV are also consistent with values reported for Fe(II)/Fe(III) oxides, while the reacted values of ∼711 to ∼712 eV are more consistent with values for Fe(III) hydroxide phases (Asami and Hashimoto, 1977).

The O 1s region (Fig. 5) of the un-reacted BnM and nZVI have a prominent contribution from a peak corresponding to an O^2−^ component at ∼530.1 eV, alongside a –OH component at ∼531.6 eV and minor H_2_O contribution at ∼533.2 eV (Biesinger et al., 2004; Manning et al., 2006). After reaction with the model Cr(VI) solution the contribution by the –OH component increased for both NPs. The BnM, however, maintained a significant O^2−^ contribution which was absent in the nZVI. The increased –OH contribution indicates the growth of a Cr(III) and/or Fe(III) hydroxide surface phase, over the original mixed valence Fe oxide (Asami and Hashimoto, 1977; Biesinger et al., 2004; Manning et al., 2006; McIntyre and Zetaruk, 1977). Upon reaction with anoxic and oxic groundwater all the samples are dominated by an –OH component, again suggesting formation of a surface hydroxide phase.

### 3.5 COPR characterization

XRF analysis of the COPR sample (Table 4) confirmed a Ca and Mg rich composition, consistent with the use of limestone and dolomite during the “high lime” processing technique (Chrysochoou et al., 2009). Fe was also present in relatively high concentrations, likely a remnant from the chromite ore. Other major components include Al, Si and Na. A high total Cr content of 3.8% (minus water and inorganic carbon) was observed by XRF. The alkaline digestion and extraction method suggested that the Cr(VI) comprised 7% of the total Cr. However, the Cr *K* edge XANES results, discussed in detail later, when subjected to linear combination fitting suggested that the samples contained 26% Cr(VI). Recent studies have highlighted underestimation of bulk Cr(VI) content by alkaline extraction of COPR due to the recalcitrance of a fraction of Cr(VI) to dissolution (Malherbe et al., 2011). The Cr *K* edge XANES is therefore assumed to represent a more robust method for bulk Cr(VI) determination. Significantly Mn, which can occur as Mn(IV) oxides that are known Cr(III) oxidizing agents (Eary and Rai, 1987), was only detected in low concentrations compared to Cr, limiting the potential for re-oxidation via this route. The high pH environment is likely to limit further the possibility of Mn(IV) oxide induced Cr(III) oxidation due to decreased solubility of reacting species (Fendorf and Zasoski, 1992).

**Fig. 6:**
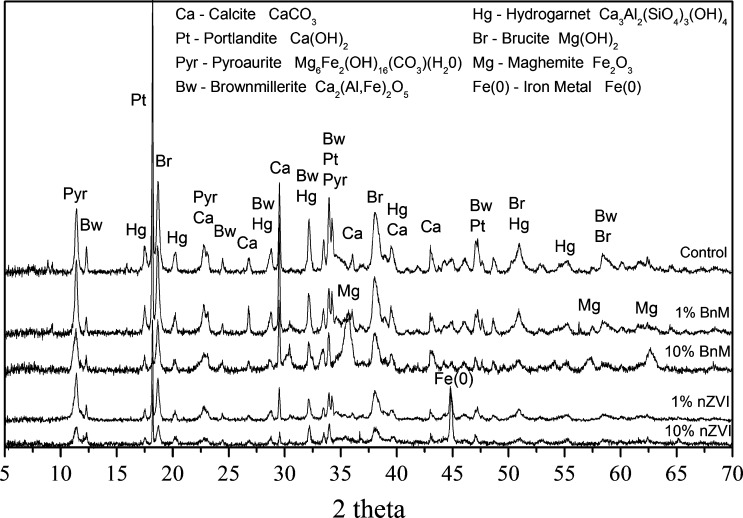
XRD analyses of the COPR control and COPR treated with 1% and 10% BnM or nZVI, after 50 days of incubation. Peaks are matched and annotated by comparisons to diffractogram from the DIFFRAC.SUITE TOPAS software database.

Remnant chromite ore was not detected by XRD (Fig. 6), although common cementitious minerals, calcite and portlandite, were detected (see Fig. 6 for formulae). Brownmillerite was present and has been inferred previously to be formed during the extraction process as a high temperature alteration product (Hillier et al., 2003). Significantly, brownmillerite has also been identified previously as containing both Cr(III) and Cr(VI) within its structure (Gibb, 1992). Portlandite, pyroaurite and hydrogarnet were also detected, all are common hydration products of brownmillerite, which is unstable in aqueous environments (Moon and Wazne, 2012). Pyroaurite is a member of the hydrotalcite group, and is closely related to the Cr(III)-containing mineral stichtite (Ashwal and Cairncross, 1997), and the Cr(VI) containing calcium aluminum chromium oxide hydrates. The latter contain chromate in brucite interlayers (Moon and Wazne, 2012), where it has the capacity for anion exchange (Geelhoed et al., 2003) and ready dissolution (Geelhoed et al., 2002), enabling leaching of Cr(VI) as chromate. The hydration product hydrogarnet has also been shown to accommodate Cr(VI) into the tetrahedral site typically occupied by the hydroxyl anion (Hillier et al., 2007). The occurrence of brucite in the sample is also inferred to be a hydration product of periclase, a common COPR mineral (Hillier et al., 2003). The absence of detectable periclase could possibly indicate a greater degree of weathering of this COPR sample compared to previous studies.

### 3.6 COPR treatment by addition of BnM and nZVI

**Fig. 7:**
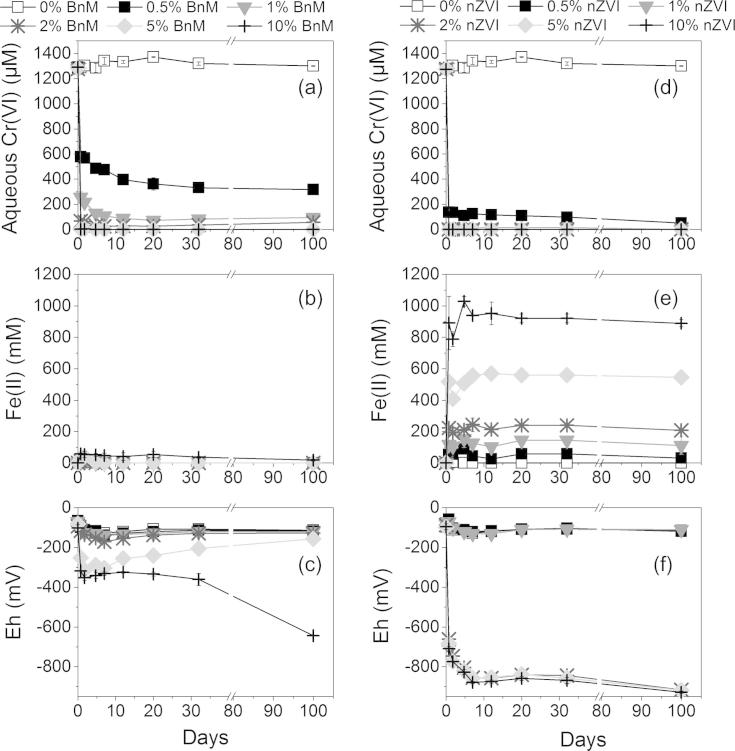
Aqueous Cr(VI) concentration and reduction potential (Eh) over time from samples of COPR amended with increasing additions of BnM (a–c), and nZVI (b–e). Error bars represent the standard deviation of the measured geochemical parameters in the duplicate microcosms. Note the break in scale between 40 and 80 days for all graphs.

The geochemical data of the COPR, treated with increasing mass % of the NP, are presented in Fig. 7. The pH of all the replicates was maintained at elevated values between 12.5 and 12.9 over 100 days of the experiment. This is inferred to be due to the strong buffering capacity of the portlandite in the COPR (Giampaolo et al., 2002).

The aqueous Cr(VI) concentration of the control (“no addition”) treatment remained relatively consistent, at 1.3 mM, throughout the experiment. The COPR sample therefore showed no capacity for natural attenuation on the timescale of this study. Increasing BnM additions removed more Cr(VI) from solution, with the 5 and 10% (by mass of field wet COPR) additions able to completely remove all soluble Cr(VI) to below the limit of detection (1 μM) within 1 day of addition. The lower additions of 1 and 2% however decreased Cr(VI) concentrations to below 0.1 mM, while 0.5% BnM equilibrated, after 100 days, at ∼0.3 mM Cr(VI). The nZVI showed a greater capacity for removal of Cr(VI), with 1% or greater bringing soluble Cr(VI) concentrations to below the detection limit (1 μM), while 0.5% was able to bring concentrations to ∼0.05 mM. The readily soluble Cr(VI) fraction of COPR can therefore be removed via the addition of ⩾5% BnM or by the lesser addition of ⩾1% nZVI, in agreement with the greater Cr(VI) removal of the nZVI seen in model aqueous solutions.

No 0.5 M HCl extractable Fe(II) was detectable by the ferrozine assay in the control treatments (Fig. 7). Readily accessible Fe(II) is unlikely to be stable in the presence of high concentrations of the oxidant Cr(VI). Addition of Fe(II)-bearing BnM to the system caused an increase in 0.5 M extractable Fe(II) concentrations after 1 day, for additions of 0.5–5% this was quickly removed to below the detection limit. The data are collectively consistent with the efficient coupling of Fe(II) oxidation to Cr(VI) reduction. The 0.5 M HCl extractable Fe(II) concentration measured with the nZVI additions maintained much higher values, which were in great excess of the Cr(VI) loadings. This is probably due to the rapid corrosion of Fe(0) to Fe(II) in the acid extraction step.

The Eh of the control (“no addition”) treatment showed a small decrease over the 100 day experiment and remained marginally negative. This decreased to a minimum of ∼−650 mV with the greatest addition of the BnM (10%), while lesser additions showed an initial decrease followed by a progressive increase. The addition of 2% and above nZVI exhibited a greater decrease to very reducing conditions, ∼−920 mV, while additions of 1% and below resembled values of the control. The addition of the higher loadings of BnM or nZVI removed all the Cr(VI) while the excess Fe(II) and Fe(0) enabled reducing conditions to prevail. At lower additions, excess Cr(VI) was able to oxidize the Fe(II) or Fe(0) and buffer the Eh towards that of the control.

**Fig. 8:**
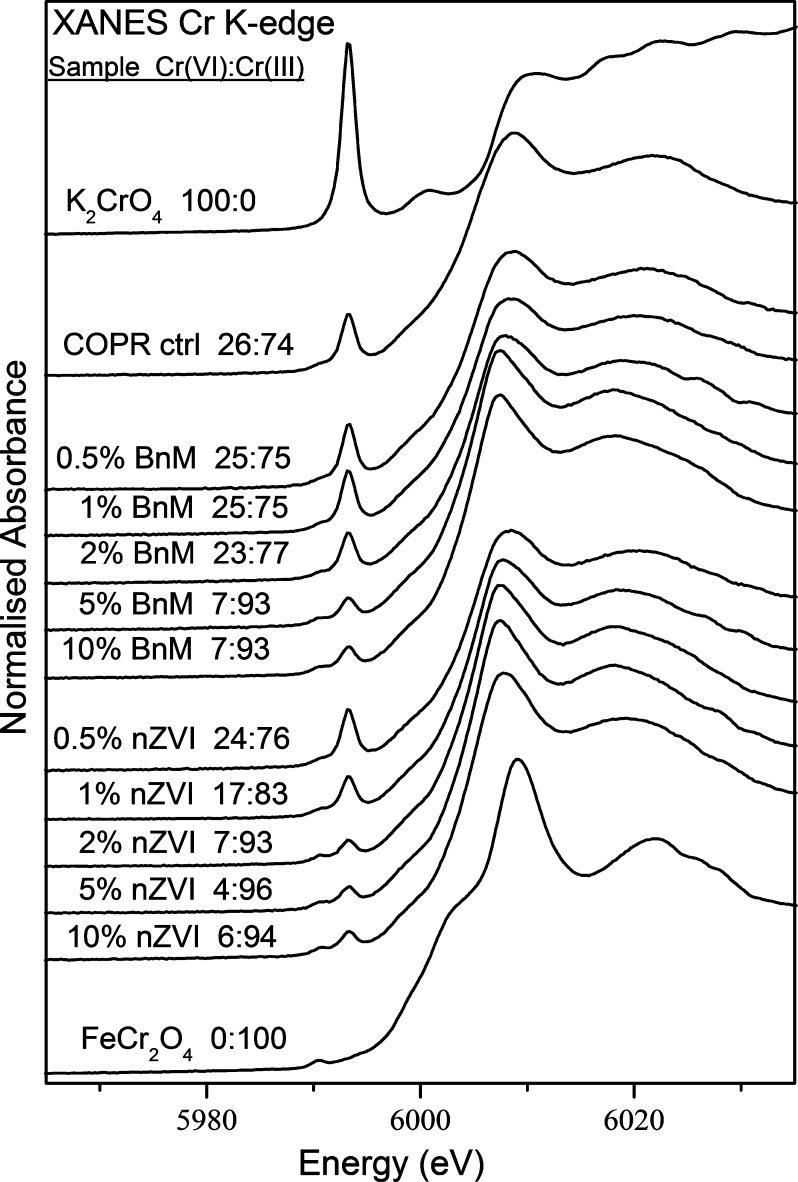
Cr *K* edge XANES spectra for the untreated COPR and COPR treated with increasing BnM and nZVI, compared to the Cr(VI) standard K_2_CrO_4_ and Cr(III) standard FeCr_2_O_4_. The values of the Cr(VI) to Cr(III) ratio from linear combination fitting are annotated on the corresponding spectra.

The Cr *K* edge spectra of the dried COPR sample was compared, by linear combination fitting, to standards for Cr(VI) (K_2_CrO_4_) and Cr(III) (FeCr_2_O_4_) (Fig. 8). The characteristic Cr(VI) pre-edge feature at ∼5996 eV (Bajt et al., 1993) was present within all COPR samples, and decreased with increasing BnM or nZVI additions. As discussed previously, the linear combination fitting calculated that 26% of the total Cr was in the Cr(VI) phase in the control COPR sample. With increasing (5% or 10%) BnM addition, this decreased to a minimum of 7% Cr(VI). While additions of 2% nZVI and greater reached a minimum range of 4–7% Cr(VI). This is in line with the findings from experiments challenged with aqueous Cr(VI), where the nZVI had a greater capacity for Cr(VI) reduction compared to the BnM. The minimum Cr(VI) value achieved, despite the presence of excess reductant in the higher addition treatments, appeared to be common throughout the treated COPR samples. As the reaction between the reactive NPs and the Cr(VI) is likely to require mobilization of the Cr(VI) into solution, further reaction is likely to be controlled by the solubility of the remaining Cr(VI). The presence of remnant Cr(VI) most likely represents the reduction of the readily soluble and reducible fraction of Cr(VI), leaving a pool of poorly soluble and therefore inaccessible Cr(VI). Previous studies using a calcium polysulfide reductant have also identified an inaccessible Cr(VI) fraction (Wazne et al., 2007), with further studies indicating a fraction of Cr(VI) that is entrapped in the poorly soluble brownmillerite phase (Chrysochoou et al., 2009).

The peak shapes of the Cr *K* edge spectra can also be used to help identify the mineralogy of the resulting Cr(III) phase. The samples exhibiting the greatest reduction appear to have a narrower main peak at ∼6008 eV located at a lower energy than the Cr(III) standard at ∼6009 eV (FeCr_2_O_4_). They also lack the shoulder feature in the edge structure at ∼6005 eV which is often characteristic of crystalline Cr(III) oxides (Whittleston et al., 2011b). The peak shapes therefore appear to resemble those of Cr(III) hydroxides, lacking the shoulder feature, in agreement with findings from XPS analyses of the NPs reacted with Cr(VI) aqueous solutions.

Upon treatment with BnM, XRD diffractogram peaks, clearly visible in the 10% addition and to lesser extent in the 1% addition samples (Fig. 6), were observed corresponding to the 311, 511, and 440 reflections tentatively assigned to maghemite but possibly occurring as an oxidized magnetite intermediate. The nZVI treated samples clearly exhibit the 110 reflection of Fe metal. Alongside this there is a minor peak possibly corresponding to the magnetite or maghemite 311 reflection. Little other mineralogical changes can be observed within the samples with no obvious Cr(III) minerals precipitated, possibly suggesting their presence as amorphous Cr(III) phases.

### 3.7 Re-oxidation of NP stabilized COPR

**Fig. 9:**
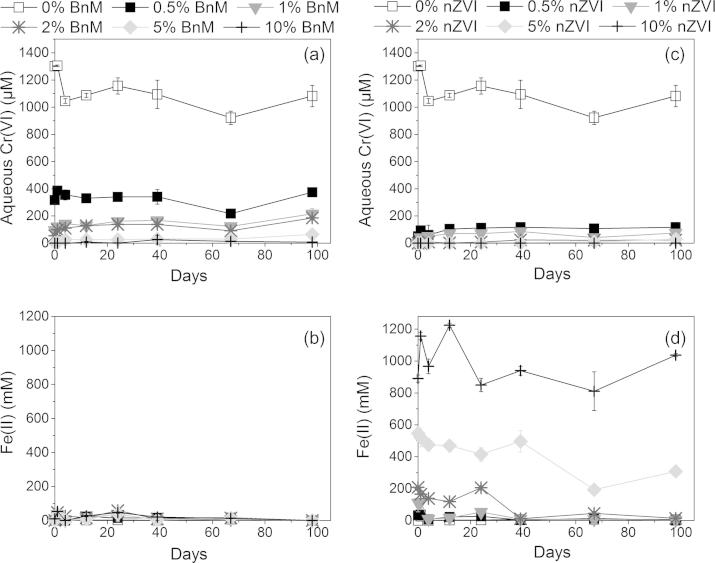
Aqueous Cr(VI) concentration (a and c) and 0.5 M HCl extractable Fe(II) (b and d), over time, of the atmospheric air equilibrated COPR treated with BnM and nZVI. Error bars represent the standard deviation of the measured geochemical parameters in the duplicate experiments.

The Eh values of the treated COPR, upon re-equilibration with air (Fig. 9), exhibited a rapid increase up to ∼−65 mV within 1 day, remaining at these values throughout the rest of the experiment. The pH of all of the replicates remained at pre-oxidation levels of 12.5–12.9 throughout the experiment. Aqueous Cr(VI) values did exhibit some minor re-mobilization upon exposure to oxygen, with all of the BnM addition replicates showing an increase in aqueous Cr(VI), although in the higher additions this is a small fraction of the original Cr(VI) values (<10%). The lower nZVI additions of 0.5–5% (by mass) showed similar low levels of Cr(VI) re-mobilization, while levels in the 10% addition remained below the detection limit (1 μM). The 0.5 M HCl extractable Fe(II) of the BnM addition replicates all remained low, with values similar to those in the stabilization experiment. The lower nZVI addition replicates of 0.5%, 1% and 2%, all showed a decrease in 0.5 M HCl extractable Fe(II) to <1 mM when exposed to oxygen, indicating air-mediated oxidation to Fe(III). The higher nZVI additions of 5% and 10% maintained higher Fe(II) concentrations over the course of the experiment, with gradual oxidation of their initially very high Fe(II) concentrations. It should be noted that observed re-mobilization of Cr(VI) upon equilibration with atmospheric oxygen can be due to either the re-oxidation of Cr(III) coupled to oxygen, or due to changes in the solubility of Cr(VI) phases upon perturbation.

## 4 Implications

The findings of this study address several aspects important when considering remediation of environmental Cr(VI) contamination using nano-scale Fe particles. Previous authors have proposed various approaches for the effective deployment of NPs *in situ*; by direct injection into the contaminant source zone or the creation of a reactive barrier to the contaminant plume (Crane and Scott, 2012; Tratnyek and Johnson, 2006). The NPs are evidently capable of stabilizing the soluble Cr(VI) fraction of the COPR, in a mock source treatment scenario, given a sufficient addition of NP. However, quantities added to the COPR, to provide an excess of the reducing Fe species, would potentially pose a challenge to effective injection. In addition, the rapid Cr(VI) removal rates, exhibited by both NPs prior to passivation, when injected into the high Cr(VI) concentrations of the COPR would potentially cause rapid Cr(III) precipitation within a zone close to the injection well. This may cause blocking of pore spaces alongside rapid passivation of the NPs themselves, potentially limiting the reactive area of influence surrounding an injection well. However, it is evident that the NPs can maintain strongly reducing conditions when in contact with COPR when added in a great excess of the Fe reductant. Careful selection of injection well emplacement would allow the formation of a reducing zone to form a barrier to both further Cr(VI) dissolution and migration.

Passivation of the NPs, preventing complete exploitation of the maximum EDC of the NP, was evidently a key limitation on their reactivity, in accordance with previous studies (He and Traina, 2005; Peterson et al., 1997). This was even more pronounced in the more chemically complex COPR contaminated groundwater, again consistent with previous findings (Cao and Zhang, 2006). The nano-scale imaging and spectroscopic data presented here offer compelling evidence to suggest this is due to the precipitation of reduced Cr(III) alongside other groundwater components to the surface of the NPs. This serves to highlight the need to consider extrinsic factors, such as chemical complexity of environmental media, when considering remediation treatment techniques. The presence of dissolved oxygen would also have a major impact on the success of NP treatment, where it readily oxidized the NPs used in this study; limiting Cr(VI) removal. Treatment of oxic groundwater using these NPs would pose a challenge, greatly increasing the quantities of reductant required for effective treatment. Significantly though, oxygen did not exhibit a capacity to re-mobilize Cr(VI) once reduced to Cr(III), suggesting this treatment technique would be stable even in changing subsurface oxygen conditions.

In addition to the extrinsic factors effecting reactivity, the intrinsic properties of the NPs are also likely to influence efficiency and susceptibility to passivation. As deployment *in situ* would preclude further amendment of the NP surface post-passivation, to regenerate reactivity, methods for maximizing the utilization of the NPs EDC should be considered. A likely solution to this problem is attempting to decrease the size of the NP, thus maximizing the surface area available for reaction. Other methods to extend the reactivity of NPs include the functionalization of the surface with Pd(0) forming a catalyst, whose reactivity can be replenished by a hydrogen donor (Chaplin et al., 2012; Coker et al., 2013; Crean et al., 2012; He and Zhao, 2008). However, by comparison to micron or granular scale alternatives the NPs tested here are highly reactive (Cao and Zhang, 2006) and potentially represent an effective treatment without further modifications.

## 5 Conclusions

This study demonstrated that both NPs investigated were capable of appreciable quantities of reductive Cr(VI) removal from alkaline solutions. Despite the nZVI removing greater total Cr(VI) quantities, the BnM was able to couple a greater proportion of its EDC to Cr(VI) reduction. Passivation of the NP reactive surface was found to be a key limitation on exploitation of the EDC of the NPs. Significantly increased passivation was noted in groundwater reacted NPs, likely due to the accumulation of Si, Ca and S, alongside Cr(III) on the NP surface. In addition to the removal of aqueous Cr(VI) from contaminated solutions both the NPs showed a capacity for the stabilization of the potent Cr(VI) source, COPR. Both treatments were able to effectively stabilize soluble Cr(VI), while bringing bulk Cr(VI) down to a minimum of 4–7%. Significantly for long term stabilization the resulting Cr(III) phases appear to be recalcitrant to re-oxidation with air, exhibiting minimal re-mobilization.

This study therefore shows the applicability of NP treatment of alkaline Cr(VI) contaminated groundwater or *in situ* source stabilization of the aqueous Cr(VI) of COPR. Importantly the reduction removal mechanism occurs at the prevalent alkaline pH of the COPR and does not require any pre-addition acidification of the media, which has previously been demonstrated to have a high acid neutralization capacity (Tinjum et al., 2008). Despite being less reactive than the nZVI the BnM achieves high Cr(VI) removal, due to its greater efficiency in coupling its available electrons to Cr(VI) reduction, and is potentially a viable alternative for reductive remediation treatments.

## Figures and Tables

**Table 1:** Chemical properties of the Cr(VI) contaminated groundwater.

Component	Method	Concentration (μM)	Concentration (mg L^−1^)
Sulfate	IC	7599	729.95
Ca	ICP-AES	5905	236.66
Chloride	IC	527	18.68
Cr(VI)	DPC Assay	321	16.69
Cr(total)	ICP-AES	342	17.78
K	ICP-AES	136	5.32
Mg	ICP-AES	8	0.19
Al	ICP-AES	5	0.13
Fe	ICP-AES	2	0.11
Mn	ICP-AES	0.02	0.01
pH	–	11.9	–

**Table 2:** Summary of Cr(VI) removal data from the batch reactivity experiments. This includes observed rates (*k*_obs_) and calculated specific rates (*k*_mass_), alongside calculated values of electrons available for donation (EDC) by the nanoparticles and the % consumed by reduction of Cr(VI).

NP	Cr(VI) reaction media	O_2_	Observed rate (*k*_obs_) (min^−1^)	*R*^2^	Specific rate (*k*_mass_) (L min^−1^ g^−1^)	Cr(VI) removed (mg g^−1^ NP)	Max EDC of NP (mol e^−^ g^−1^ NP)	e^−^ consumed by Cr(VI) reduction (mol e^−^ g^−1^ NP)	% e^−^ consumed by Cr(VI) reduction
BnM	Model Cr(VI)	Anoxic	0.71 × 10^−3^	0.95	0.94 × 10^−3^	32	4.32 × 10^−3^	1.85 × 10^−3^	43
	Cr(VI) GW	Anoxic	1.01 × 10^−3^	0.90	1.50 × 10^−3^	24		1.38 × 10^−3^	32
		Oxic	–	–	–	7		0.40 × 10^−3^	9

nZVI	Model Cr(VI)	Anoxic	0.50 × 10^−3^	0.91	1.90 × 10^−3^	79	40.29 × 10^−3^	4.56 × 10^−3^	11
	Cr(VI) GW	Anoxic	0.72 × 10^−3^	0.94	3.20 × 10^−3^	40		2.31 × 10^−3^	6
		Oxic	–	–	–	15		0.87 × 10^−3^	2

**Table 3:** Surface analysis data of the un-reacted and reacted NPs. Note GW represents groundwater reacted samples.

	Cr(VI) reaction media	Oxygen	XAS	XPS (atomic%)					XPS		XMCD	
			% Cr of Fe	% Fe	% Cr	% Ca	% S	% Si	% Fe(II)	% Cr(III)	% Fe(II)	T_d_:O_h_
BnM	Unreacted	Anoxic	–	100	–	–	–	–	30	–	27	0.50
	Model Cr(VI)	Anoxic	11.1	71	29	–	–	–	25	85	26	0.50
	Cr(VI) GW	Anoxic	–	26	12	30	7	25	23	93	–	–
		Oxic	–	36	8	26	7	23	6	81	–	–

nZVI	Unreacted	Anoxic	–	100	–	–	–	–	24	–	26	0.49
	Model Cr(VI)	Anoxic	15.3	53	47			–	27	98	25	0.49
	Cr(VI) GW	Anoxic	–	26	7	28	7	32	18	85	–	–
		Oxic	–	39	8	23	6	24	8	88	–	–

**Table 4:** XRF results of major chemical component analysis obtained from the untreated COPR sample, expressed as common oxides. Data not corrected for loss on ignition.

Major chemical component	%
CaO	31.3
MgO	30.2
Fe_2_O_3_	14.3
Na_2_O	6.4
Al_2_O_3_	6.0
SiO_2_	5.1
Cr	3.8
SO_3_	1.9
Mn	0.2
