# Supplementary material for: Biogenic nano-magnetite and nano-zero valent iron treatment of alkaline Cr(VI) leachate and chromite ore processing residue
Source: Appl Geochem. 2015 Mar;54:27–42. doi: 10.1016/j.apgeochem.2014.12.001 (PMC4461148; doi:10.1016/j.apgeochem.2014.12.001)
Supplement: Supplementary data 1 [file mmc1.docx]

Supporting Information (SI) to

Biogenic nano-magnetite and nano-zero valent iron treatment of alkaline Cr(VI) leachate and chromite ore processing residue

Mathew P Watts^1^, Victoria S. Coker^1^, **Stephen A. Parry^2^, R**ichard. A. D Pattrick^1^, Russell A. P. Thomas^3^, Robert Kalin^4^& Jonathan R. Lloyd^1^

^1^ School of Earth, Atmospheric and Environmental Sciences and Williamson Research Centre for Molecular Environmental Science, The University of Manchester, Manchester, M13 9PL, U.K. ^2^ Diamond Light Source, Chilton, Didcot, Oxfordshire, OX11 ODE, U.K. ^3^ Parsons Brinckerhoff, Queen Victoria House, Redland Hill, Bristol, BS6 6US, U.K. ^4^ Department of Civil and Environmental Engineering, University of Strathclyde, Glasgow G1 1JQ, UK

* Jon.Lloyd@postgrad.manchester.ac.uk





Fig. SI 1. XRD diffractograms of un-reacted biogenic magnetite (BnM) in black and nano zero valent iron (nZVI) in grey.





Fig. SI 2. Pseudo-1^st^ order reaction fitting for Cr(VI) removal from a model Cr(VI) pH 12 solution (a), when treated with 0.75 g L^-1^ BnM or 0.25 g L^-1^ nZVI, and Cr(VI) contaminated groundwater (b) when treated with 0.66 g L^-1^ BnM or 0.22 g L^-1^ nZVI.
